# Experiences from national biodiversity data infrastructures in Europe: advancing data integration and community engagement

**DOI:** 10.1093/biosci/biag032

**Published:** 2026-05-04

**Authors:** Birgit Gemeinholzer, Aletta Bonn, Barbara Ebert, Sarah Fischer, Anton Güntsch, Jörg Holetschek, Veronika A Johansson, Martin Friedrichs-Manthey, Juliane Röder, Christoph Schomburg, Margret Steinthorsdottir, Elaine van Ommen Kloeke, Hugo J de Boer

**Affiliations:** University of Kassel, AG Botany, Kassel, Germany; Department Community Ecology, Helmholtz Centre for Environmental Research—UFZ, Halle, Germany; Institute of Biodiversity, Friedrich Schiller University Jena, Ecology and Evolution, Jena, Germany; German Centre for integrative Biodiversity Research (iDiv) Halle-Jena-Leipzig, Leipzig, Germany; GFBio—Gesellschaft für Biologische Daten e.V., Bremen, Germany; Research Institute for Farm Animal Biology (FBN), Dummerstorf, Germany; Botanic Garden and Botanical Museum Berlin, Freie Universität Berlin, Berlin, Germany; Botanic Garden and Botanical Museum Berlin, Freie Universität Berlin, Berlin, Germany; Department of Bioinformatics and Genetics, Swedish Museum of Natural History, Stockholm, Sweden; Institute of Biodiversity, Friedrich Schiller University Jena, Ecology and Evolution, Jena, Germany; German Centre for integrative Biodiversity Research (iDiv) Halle-Jena-Leipzig, Leipzig, Germany; Staff Office for Research Data Management, Marburg University, Marburg, Germany; University of Kassel, AG Botany, Kassel, Germany; Department of Bioinformatics and Genetics, Swedish Museum of Natural History, Stockholm, Sweden; Naturalis Biodiversity Center, Leiden, Netherlands; Natural History Museum, University of Oslo, Oslo, Norway

**Keywords:** Global Biodiversity Framework (GBF), national data centers, stakeholder management, sustainable development goals, long-tail data

## Abstract

The urgent need for comprehensive biodiversity data is driven by rapid biodiversity loss due to human activity. Drawing on insights from established national biodiversity data infrastructures in Europe, this article highlights eight key considerations for strengthening national data infrastructures—particularly in enhancing stakeholder engagement through improved data availability and accessibility. We emphasize the importance of collaboration among diverse stakeholders to enhance data sharing and integration. By utilizing technological advancements, implementing international standards for data interoperability under FAIR (Findable, Accessible, Interoperable, and Reusable) conditions, establishing robust communication strategies, and providing necessary training and legal guidance, these infrastructures can support effective data mobilization. Furthermore, promoting a culture of collaboration among stakeholders enhances the quality and applicability of biodiversity data for scientific research, policy, and conservation efforts. Our recommendations aim to ensure that national biodiversity data infrastructures effectively contribute to achieving the UN Global Biodiversity Framework targets and Sustainable Development Goals by encouraging strong partnerships and efficient data management practices.

## Introduction

Human activities are driving significant changes in biodiversity worldwide (Dornelas et al. 2014, Keil et al. 2015, Kaarlejärvi et al. 2021, Turner et al. 2023). In order to inform decision-makers about resource management and conservation planning, biodiversity data are essential for understanding the effects of climate change and anthropogenic influences on environmental changes (Parr et al. 2002, Thornhill et al. 2021, Peterson et al. 2023). There is a broad range of interest groups, collecting biodiversity data, providing tools and services for making data accessible and interoperable, or being interested in reusing data. These include non-professional researchers, scientists, experts and professionals, nature conservationists, government and policymakers, data infrastructure, and technology providers, as well as the private sector and industry. However, important biodiversity data only can be obtained if stakeholders are willing to share data, collaborate, and connect around a common vision (Kühl et al. 2020). Hence, it is crucial to develop robust solutions and effective strategies for providing and integrating biodiversity data by involving a diverse range of partners who are willing to collaborate and share expertise (Güntsch et al. 2024).

Biodiversity data are collected across diverse topics, and are often heterogeneous, fragmented, and span different spatial and temporal scales. Data vary in type, including occupancy, distribution, observational and experimental data; physical collections; molecular and organismic data; remotely sensed and laboratory-based data. They also differ in levels of quality assurance and the availability of metadata. In recent years, sophisticated technological innovations have emerged to improve the speed and capacity of sensor-driven automated biodiversity monitoring data (Kosmala et al. 2016, McKinley et al. 2017, Pocock et al. 2017, Thornhill et al. 2021, van Klink et al. 2022), experimental and assessment data (e.g., Boyd et al. 2021), multimedia, sequence-related information, or remotely sensed data for biodiversity-related challenges (e.g., Berendsohn et al. 2011, Bird et al. 2014, Geldmann et al. 2016). Furthermore, the ongoing digitization of historical and current data is improving the biodiversity data landscape by enabling the analysis of changes in biodiversity patterns over longer periods of time. Although a variety of actors generate a wealth of biodiversity data, a variety of data infrastructures have been developed and maintained by research organizations, expert groups, natural history museums, and government agencies, with overlapping roles, resources, challenges, and responsibilities in some cases (e.g., table 1).

**Table 1:** Origin of stakeholders with their motivations, challenges, and incentives to participate in collaboration in joint biodiversity data infrastructures

Origin of stakeholders	Motivation	Benefits	Challenges to participate	Incentives for participation
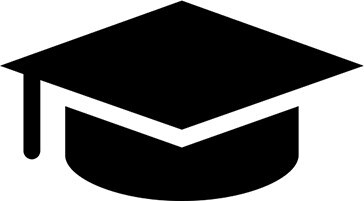 Research institutions, federated research infrastructures, universities	Scientific interest, sharing motivation, obligation to conduct FAIR* research data management	Access to and provision of biodiversity data, increasing the number of publications and citations, meeting requirements of external funders	Limited resources and support, concern of losing, scientific independence, lack of research data management training	Access to funding sources and large-scale data, increased visibility, and recognition of research output, such as publications
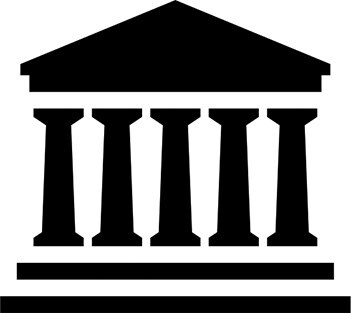 State authorities and governmental agencies	Statutory obligation and reporting duties	Access to comprehensive, standardized, quality-controlled data and metadata	Limited resources, understaffing, political conflicts, concern of losing sovereignty, varying regional responsibilities, and different reporting tools	Closing existing gaps (spatial, temporal, taxonomic), harmonizing data and data exchange, benefit from collaboration
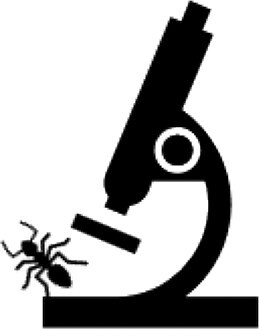 Natural history museums, collections, data centers, national park authorities	Scientific interest, sharing motivation, obligation to conduct FAIR* research data management	Support in data management, access to analytical and statistical expertise, public recognition	Limited resources concerning funding and staff	Increased visibility, access to new funding sources, influence on policy, enablement of interinstitutional and international networking activities
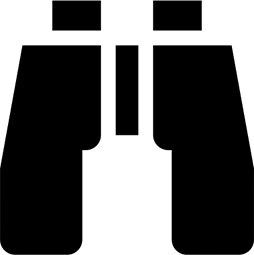 Non-profit biodiversity data holders, natural history societies, biological field stations, participatory experts	Intrinsic motivation	Community building, exchange with like-minded people, impact at the science–policy interface	Limited resources for maintenance of biodiversity data infrastructures, networking activities, and knowledge, reluctance to share data arising from privately funded initiatives	Support with data standards, software, and networking solutions as well as access to analytical and statistical expertise. Increased value through collaboration and enhanced visibility
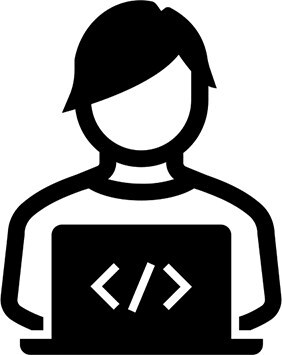 Private sector companies	Improved public reputation, fulfilment of reporting duties	Access to data, additional information, monetary and marketing benefit when sharing data	No financial benefit in sharing data. Risk of losing competitive advantage	Access to available, previously collected data reduces costs, expanding contact networks, increased visibility

* FAIR: Findable, Accessible, Interoperable and Reusable in accordance to Wilkinson and colleagues (2016).

Currently, various data infrastructures have been established for specific organizational or data-related contexts; some are broad in scope, whereas others are highly specialized and represent only a niche, narrow slice of the multidimensional space of biodiversity data (Bingham et al. 2017). Various biodiversity-related data infrastructures have been initiated at the national level. These offer high-quality, curated, and expert-validated data, and thus trustworthy resources on specific biodiversity topics, often with direct links to the data providers, some of which are expert-curated resources with often high-quality, carefully validated data. However, many data infrastructures are only partially consolidated because they are not part of an institutional and sustainable data strategy. As a consequence, these data do not adequately contribute to the need for monitoring, decision-making, or large-scale biodiversity assessments.

To meet the growing demand for biodiversity data, local and often isolated data platforms must be integrated into globally accessible services developed through collaborative efforts (Güntsch et al. 2024). Biodiversity data infrastructures form the foundation for multiscale data availability, enabling the sharing of unprecedented volumes of data, resources, and expertise across a wide range of biodiversity initiatives—whether taxon-specific, general, or specialized—worldwide. The rapid expansion of network infrastructures, enhanced computing power, and ongoing standardization of data formats and access protocols—for example, in the biodiversity community, led by organizations such as Biodiversity Information Standards (TDWG)—have facilitated the development of a diverse range of advanced and reliable international data services (Güntsch et al. 2024). These services include platforms for recording observations, aggregation tools that provide consistent access to distributed biodiversity data, and systems for accessing taxonomic backbones and ontologies. Biodiversity data infrastructures must be consistently updated, tailored, and expanded by experts to meet the diverse needs of users and federated biodiversity data systems (Waterhouse et al. 2024). This adaptability is vital for effective biodiversity data management and demands that specialists are not only proficient in the latest software and hardware but also deeply knowledgeable about evolving standards and robust data exchange systems. Ongoing support and maintenance are vital to facilitate continuous data mobilization and updates, as well as to promote the exchange of training, expertise, and legal guidance, ensuring the long-term sustainability and accessibility of these invaluable resources. Thus, aggregating biodiversity data and consolidating infrastructures relies on both community-specific and international networks [e.g., the BKH (Biodiversity Knowledge Hub: https://biodiversityknowledgehub.eu/), OBIS (Ocean Biogeographic Information System: Ocean Biodiversity Information System), and GBIF (Global Biodiversity Information Facility: https://gbif.org), among others]. This effort is needed for the seamless integration of biodiversity data across various platforms and networks, which is further supported by initiatives like the Group on Earth Observations Biodiversity Observation Network (GEO BON: https://geobon.org, Pereira et al. 2013).

Recently, numerous countries have initiated comprehensive activities and infrastructures to enhance the collaborative provision of biodiversity data from various national sources (e.g., Güntsch et al. 2024). This is essential as most activities and policies in action happen at the local scale. In order to make data-driven decisions, data need to be generated and shared on the local, regional, national, and international level. Key contributions from national biodiversity data infrastructures come from national authorities, such as those in Ireland, Sweden, Norway, Australia, the United Kingdom, South Africa, the United States, Brazil, Mexico, and the Netherlands; academic institutions in Colombia and Ireland; non-profit organizations like the National Biodiversity Network (NBN) in the United Kingdom; and project-funded initiatives like iDigBIO in the United States and NFDI4Biodiversity in Germany (Glöckner et al. 2020, see table 2). All of these actors are now increasingly working together to strengthen global biodiversity data mobilization efforts. For example, the Swedish Biodiversity Data Infrastructure (SDBI), the largest collection of freely available biodiversity data in Sweden, aggregates biodiversity data from 241 different data sources from 107 collections and 31 institutions (28 January 2026) and makes it available and usable online. Many countries, however, lack coordinated national biodiversity data infrastructures or collaborative organizational structures (Walters and Scholes 2017, Pacifici et al. 2018, Hobern et al. 2019, Schulman et al. 2021, Rosa et al. 2021, Güntsch et al. 2024, and others) to facilitate the sharing of data, resources, and expertise among manyfold local, taxa-specific, or specialized biodiversity data initiatives.

**Table 2:** Examples of national biodiversity data infrastructures with their data types, interest groups, and organization types (centralized/decentralized) whose direct or published findings have been included in our analysis.

Infrastructure, country of origin	Coverage of data types	No. of partner organizations	Partner community	Functionalities/ stakeholder support	Organization structure
Authoritative and Rapid Identification System for Essential biodiversity information/ARISE (Netherlands)	Species information Occurrence Images Sounds Telemetry Molecular data AI models	4	Researchers NGOs Policymakers Industry Authorities Participatory science (community science)	Species reference databases Digital sampling tooling & kits high throughput imaging for specimens DNA barcoding & genome skimming Bioinformatic tools AI model repository Data management for harmonizing data Online training modules Newsletter	Centralized
Atlas of Living Australia (Australia)	Species occurrences Species information Molecular data Images Sound Social and economic monitoring Maps	4	Researchers Practitioners Policymakers Participatory science NGOs Community and education groups	Data provisioning Tool provisioning Providing species profiles Software environments Ecosystem and habitats portal Red Lists Invasive species Cultural heritage Data management for harmonizing data	Centralized
Biodiversity Ireland (Ireland)	Species occurrences Maps Biodiversity inventory National biodiversity indicators	4	Researchers Practitioners Policymakers Participatory science NGOs Authorities	Data provisioning Providing species profiles Data submission tool Training Ecosystem and habitats portal Red Lists Invasive species Data management for harmonizing data	Centralized
Integrated Digitized Biocollections (United States)	Species occurrences Paleontological data Maps	6	Researchers Practitioners Policymakers Participatory science NGOs Authorities	Data provisioning Tool provisioning Workshops and training material Conferences Helpdesk Data management for harmonizing data	Distributed
National Biodiversity Network (United Kingdom)	Species occurrences Monitoring initiatives National biodiversity indicators Maps	>200	Researchers Practitioners Policymakers Participatory science NGOs Authorities Monitoring programs	Data provisioning Tools provisioning Newsletter Conference organization Helpdesk Data management for harmonizing data	Centralized
Nationale ForschungsdatenInfrastruktur4Biodiversity (Germany)	Species occurrences Species information Molecular data Ecological data Images Maps	>50	Researchers Policymakers Participatory science NGOs Authorities IT specialists	Data provisioning Tool provisioning Training Legal advice Conference organization Infrastructure availability Cloud environment Helpdesk Portal Archiving	Distributed
Norwegian Biodiversity Information Centre (Norway)	Species occurrences Species information Maps Red Lists Invasive Species Ecosystem and habitats information	1	Researchers Policymakers Participatory science NGOs Authorities	Data provisioning Tool provisioning Portal Archiving	Centralized
Sistema de información sobre Biodiversidad de Colombia (Colombia)	Species occurrences Molecular data Monitoring Images Remote sensing National biodiversity indicators Maps	>100	Researchers Practitioners Policymakers Participatory science NGOs Authorities Farmers	Data provisioning Providing species profiles Portal Archiving	Centralized
Sistema Nacional de Información sobre Biodiversidad de México	Species occurrence Species information Images Ecological data Maps Remote Sensing National biodiversity indicators Ecosystem and habitat information Cultural heritage Tourism	125	Researchers Practitioners NGOs Community and education groups	Data provisioning Tool provisioning Training Legal advice Conference organization Infrastructure availability Cloud environment Helpdesk Portal Archiving	Centralized
South African National Biodiversity Institute (South Africa)	Species occurrences Species information Molecular data Images Sounds Biodiversity inventories Maps Invasive species	>100	Researchers Practitioners Policymakers Participatory science NGOs Community and education groups Tourism	Data and links provisioning Tool provisioning Training	Centralized
Swedish Biodiversity Data Infrastructure (Sweden)	Species occurrences Molecular data Archaeological data Biotelemetry/biologging data Monitoring initiatives National biodiversity indicators Maps	12	Researchers Practitioners Policymakers Participatory science NGOs Authorities Monitoring programs	Data provisioning Tool provisioning Workshops and training Conference organization Helpdesk	Distributed

Although several publications have addressed the technical challenges of networking and aggregating biodiversity data, less attention has been given to the diverse and wide-ranging stakeholders whose involvement makes managing and communicating both centralized and decentralized biodiversity data infrastructures a persistent challenge (Adams et al. 2002, Alphandéry and Fortier 2010, Turnhout et al. 2014, Strandburg et al. 2017, Sterner et al. 2020). So far, there are only a few examples and little guidance to support stakeholder integration at different levels. Despite widespread willingness and awareness among stakeholders to share biodiversity data and recognize its added value, the specific needs for effective data sharing—encompassing legal and technical aspects, biodiversity data collection, annotation and synchronization, analysis, and visualization—vary widely, posing significant challenges for network activities.

This paper reflects current developments in national biodiversity data infrastructures and focuses on stakeholder engagement strategies in the shared provision and use of open and FAIR (Findable, Accessible, Interoperable, and Reusable) biodiversity data (Wilkinson et al. 2016) and data infrastructures, herby supporting the idea of the Global Open Research Commons (Treloar and Woodford 2025) in biodiversity research with a focus on stakeholders’ networking and ecosystem governance (Yang and Hu 2026). Based on a strategic framework, provided by Berndtsson and colleagues (2018) for data-driven organizations, we adopted and extended their transformation goals (1. what to change, 2. how to measure progress, and 3. how to change) by placing a stronger focus on stakeholders’ requirements, concerns, and demands as well as their management and communication needs. Building on our experience of different national biodiversity data infrastructures, we identify eight key components for a successful community and stakeholder engagement in comprehensive biodiversity data infrastructures. These can be applied both for small-scale data aggregation to large, multisource networks for high-quality biodiversity data for participatory experts, scientists, policymakers, as well as basic and applied research.

## Defining collaboration and benefits among biodiversity data holders

The biodiversity data landscape consists of a network of very diverse actors who contribute different relevant data, expertise, problems, and motivations as well as various types of information, tools and services, infrastructures, training material and designs, among others, as a non-negligible asset (Henry et al. 2008, Pereira et al. 2010, Kühl et al. 2020). Information on biodiversity highly depends on actors' diversity (Hobern et al. 2019). Thus, the aim is to connect main actors in biodiversity data infrastructures with developed networking solutions with the independent efforts of other actors with shared or similar needs (tables 1 and 3). This results in “long-tail” data, which combines various heterogeneous data collections to generate knowledge, and which requires collaboration, management, and institutional support, for example, in the biodiversity communities exemplified by global biodiversity initiatives such as GBIF and OBIS among others (Stahlman and Kouper 2024). In this context, collaboration is defined in such a way that mutual benefit between stakeholders is created through cooperation. Here, stakeholders are defined as a group of people with the aim of jointly mobilizing, aggregating, providing and networking biodiversity data, providing support, technical solutions, training, and exchange formats as well as legal advice through a common networked working environment. All stakeholders are willing to collaborate, share data and knowledge, and, wherever possible, support others in their biodiversity data management activities. For example, data providers may collaborate with scientific IT centers and IT developers to implement data management solutions and/or mobilize data for scientific use. As a result, collaboration encompasses all solutions that facilitate efficient stakeholder collaboration. Customization and adaptability are essential components of successful collaboration, and different partners may have unique data, software, hardware, requests, requirements, problems, and solutions.

**Table 3:** Overview of main stakeholder tasks requiring support from national biodiversity data infrastructures, including group size, effort, automation potential, expert knowledge needs, and training demands.

Requests for biodiversity research data infrastructure support in…	Group size	Contact frequency	Automation potential	Expert knowledge need	Training demands
research data management	Large–medium Large	Not frequently	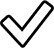 High	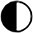 Medium	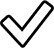 High
data and metadata use	Large–medium Large	Not frequently → frequently	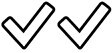 High	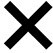 Low	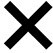 Low
archiving of data and metadata	Large–medium Large	Not frequently → frequently	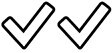 High	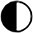 Medium	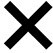 Low
training and education	Large	Not frequently → frequently	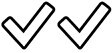 High	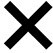 Low	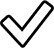 High
the use of analysis tools	Medium–large	Not frequently	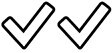 High	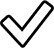 High	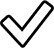 High
services (e.g., legal advice)	Medium–large	Not frequently	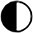 Medium	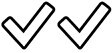 High	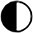 Medium
cloud storage and work space	Medium–large	Not frequently → frequently	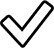 High	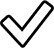 High	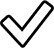 High

Connecting previously unconnected data holders and fostering collaboration may ensure far greater availability of biodiversity data beyond the capacity of any single infrastructure or data holder in real-world conditions. In addition, biodiversity data stakeholder alliances act as resource providers and expert networks, adding value to the community by providing a single point of contact for support, training, and education. A key benefit of collaboration must be to encourage the adoption of recognized standards, tools, and workflows within respective fields, without rendering the original or local data infrastructures obsolete. Because there is no one-size-fits-all solution, collaboration in biodiversity data networking must be both versatile and adaptable allowing users and groups to adapt and engage in a variety of ways (Coyle 2024). The collaborative approach and cooperation with various stakeholders facilitate the collective needs of the group, while also accommodating, whenever possible, the individual needs of each member. Therefore, it is important to emphasize that collaboration serves as a way to find the best solution, with the highest level of trust, quality standards, and requirements for each specific area and community. It also fosters an environment where joint efforts are not only recognized as beneficial but actively pursued to maximize the benefits for all stakeholders. This also raises stakeholders' awareness to connect with their activities and thereby realizes that they are part of a group whose individual visibility is maintained but also promoted by a critical mass of users. In this way, stakeholders recognize that this networking and infrastructural consolidation means that the whole is greater than the sum of its parts (Kühl et al. 2020). This requires a strong but also flexible networking approach across stakeholders, knowledge domains, and institutions (Neßhöver et al. 2015), where the alliance should be inclusive and responsive to potential future ameliorations and needs, allowing for the involvement of further stakeholders as the collaboration evolves, while also considering the interests of users who may benefit from data availability (Hobern et al. 2019).

## Conceptualizations of stakeholder communities for collaboration

Collaborative stakeholder activities are generally based on a prior conceptualization of actors with common interests or expertise who are willing to work together, share knowledge, and influence each other through their activities (Garfield 2020). The contextualization of actors of national biodiversity data infrastructures can be predefined and fixed or the result of a process. However, a prerequisite is the coming together of like-minded partners and organizations willing to develop a common vision and understanding to work toward a well-organized structure with common standards, forming the base of provision of well-developed tools and services. This shared vision is important for both active operation and further development of the infrastructure toward a highly complex participatory network system, where stakeholders are involved not only in the transmission and use of data, but also in the provision and development of tools and services (e.g., NFDI4Biodiversity, SBDI, ARISE, and others).

**Figure 1:**
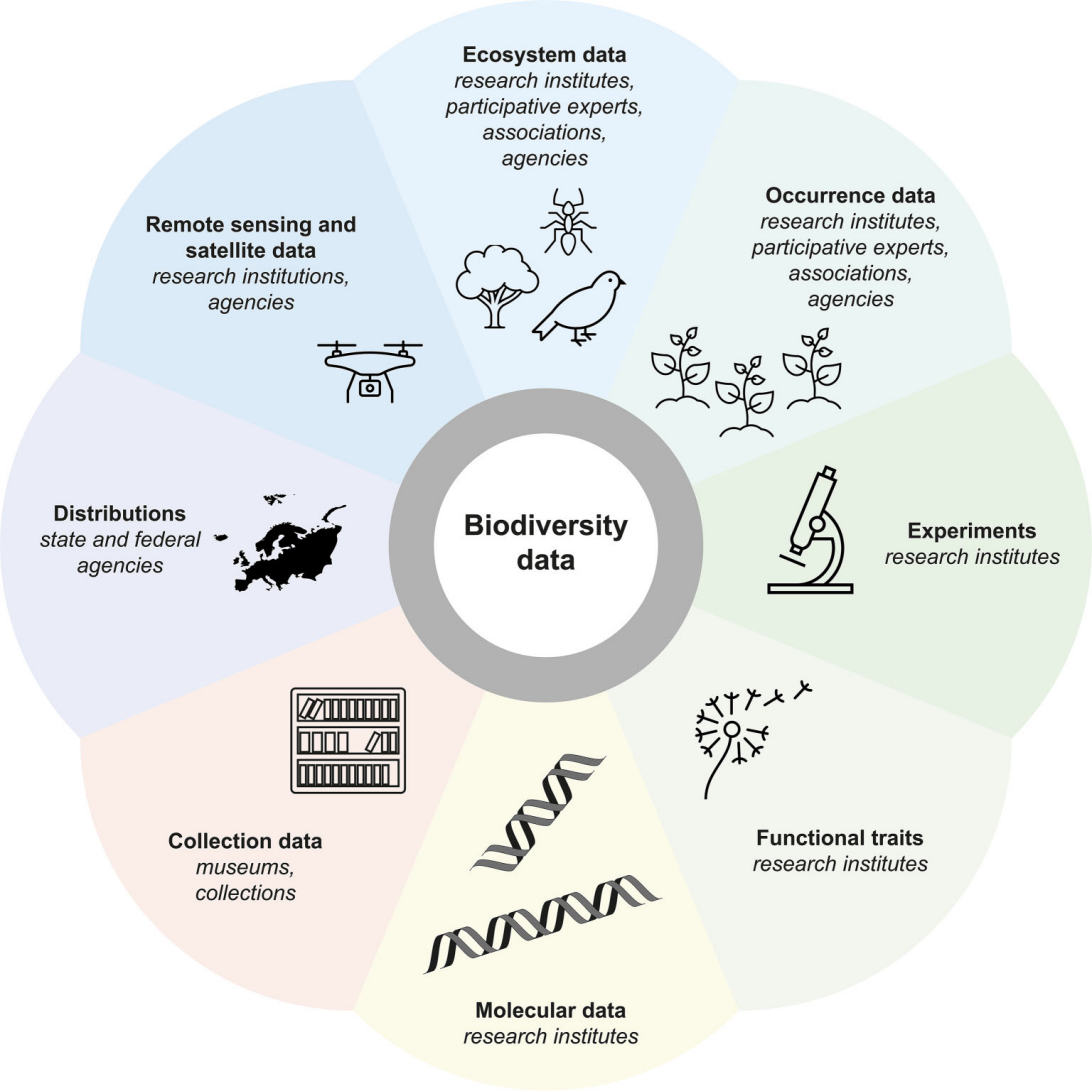
Examples of biodiversity data and their potential origin from different actors.

Biodiversity-related data stakeholders are generally data holders or representatives of user groups who collect, coordinate, analyze, and visualize data. They are also involved in software and hardware development, interoperability and accessibility, storage, data management, standardization, education, and training. However, actors can also be conceptualized on the basis of the different types of data they provide. This, for example, can be occurrence data, which enriches the understanding of biodiversity and is often contributed by participatory experts, governmental agencies, natural history collections, and research institutions, whereas satellite data most often are provided by research institutions and agencies (figure 1). On the other hand, biodiversity data holders can be classified on the basis of their associated backgrounds or institutions, such as being part of research institutions and universities, natural history collections, governmental authorities, natural history societies, biological field stations, the private sector, or others (table 1). The SBDI consortium in Sweden, for example, is primarily composed of stakeholders from universities, natural history museums, and scientific institutions, whereas the ARISE in the Netherlands has four research institution partners as headquarters that build a national research infrastructure for the wider community of more than 200 professional and non-professional experts. The NBN consortium in Scotland connects more than 200 large and small organizations as a dedicated community of individuals passionate about wildlife data.

To capture the perspectives of diverse stakeholders, personas and user stories can be developed. This approach helps illustrate the value that specific aspects of the infrastructure or service may offer to different user types. For instance, in NFDI4Biodiversity, personas were defined to represent the needs of four different fictitious but life-like individuals, whose interests and needs cover the consortium’s target groups. A nature lover and conservationist, a postdoctoral researcher, an institute director, and a data manager were selected (www.nfdi4biodiversity.org/en/personas/), and the benefits of the national biodiversity data infrastructure were evaluated through their different perspectives. In routine operations, the importance of personas probably fades into the background, and issues with higher stakeholder priority take precedence, yet the initial realization that understanding who the activities should serve is important for the design and development.

Another method for conceptualization is to design key categories, based on the stakeholders’ primary interests in engaging with the biodiversity data network: (1) data mobilization; (2) enhancing data management practices; (3) advancing standardization, storage, and publication; (4) training, legal issues, and capacity-building; and (5) improving analysis, integration, and accessibility. These categories are more based on stakeholders’ expertise or motivation, including participatory experts, ecologists, researchers, rangers, public employees, (bio-)informaticians, teachers, and trainers. An advantage is that this reflects that some actors offer extensive data management expertise: they have decades of experience in biodiversity data collection, storage, or export of datasets that are standardized and FAIR, with sophisticated databases, and international networks. These tasks depend on experienced (bio-)informaticians who are familiar with robust and state-of-the-art tools, global developments, standards, interoperability, and digital networking strategies. On the other hand, it reflects how other stakeholders have developed simple database solutions that are robust, accepted, and maintained by the expert community, but would be willing to change to more flexible, interoperable systems. However, during collaboration, these distinctions probably diminish and other motivations to continue working together take center stage. Thus, these categories are not rigid and may overlap and change during the process, as stakeholders generally have multiple roles and cannot be clearly assigned to one category. Overlapping and changing roles, work programs, and responsibilities at different levels make it difficult to conceptualize all parties involved in addressing the complex challenges. Nonetheless, these categories provide initial insights into understanding stakeholders' initial motivations. This is also supported by the research on stakeholder theory, which adds that there are significant differences between stakeholders' perceptions of their own interests and the expectations placed on them (Bryson 2004), which requires in-depth discussions to understand (Calton and Payne 2003). However, when it comes to solving individual problems related to detailed issues, stakeholders readily assign themselves to one group or another, which may result in lasting and mutually beneficial relationships (Maak 2007, De Gooyert et al. 2017).

## Stakeholders’ concerns and motivations

The success of stakeholder integration in national data infrastructures for biodiversity data depends on the strong commitment and determination of partners (table 1). Motivations vary but include funding, good scientific practice, increased visibility through data publication, networking and exchange between expert groups, access to tools and services, added value through increased data availability, international networking, and policymakers or reporting obligations. Stakeholders' benefits and incentives to connect with national biodiversity data infrastructures include new ideas for shared management and analysis of biodiversity data in cloud systems, data transformations with semantic tools, the use of temporary object storage, or the operation of own applications on servers in multicloud systems. However, intrinsic motivations, like bending the curve of the biodiversity crisis or supporting positive actions for nature and science, are sometimes the driving force for collaboration (e.g., many participatory experts). The key to effective stakeholder engagement is authentic and relevant involvement (Crowley et al. 2023). We recommend reconciling the different motivations, objectives, expectations, and obligations to reconcile the different motivations, objectives, expectations, and obligations in aggregating and networking biodiversity data by reducing the risk of unintended conflict or duplication of effort. This can be achieved by bringing people together through conferences and networking activities. Suppose continuous stakeholder involvement is a prerequisite for the infrastructure process, as is the case for NBN, NFDI4Biodiversity, and ARISE. In that case, there must be a balance between participation and offering effective practices for building and strengthening cooperative relationships, such as conducting training events, summer schools, or providing contacts through a helpdesk. Sterling and colleagues (2017) stated that the degree of participation matters, and they summarized in studies in the field of biodiversity conservation that more collaborative, participatory processes led to better results (Beierle 2002, Reed 2008, Brooks et al. 2013), whereas reliance on predominantly low-quality participation rather than active involvement may reduce effectiveness (Pollini and Lassoie 2011, Minter et al. 2014). However, some stakeholders act more like passive observers than active drivers of national biodiversity data infrastructures. Reluctance to participate may stem from financial constraints, personnel bottlenecks, disruptions to ongoing project activities, political or funding-related concerns, or the additional effort required for further activities. Concerns and legal requirements must be taken into account (e.g., that open data may endanger sensitive species and their habitats or be used for commercial purposes without benefit sharing) (Griffiths et al. 2015, Fox et al. 2019, Turner et al. 2022). Also, this highlights the need for a network or ecosystem design that is characterized by trust, modularity, and transparency (Jacobides et al. 2018), which is flexible enough to accommodate actors' roles and responsibilities and integrate essential elements of their respective work programs, services, and interdependencies. Trust and credibility are key to ongoing stakeholder engagement, which requires continuity. The FAIR (Wilkinson et al. 2016) and CARE principles (Collective Benefit, Authority to Control, Responsibility, Ethics, Carroll et al. 2020) as well as TRUST (Transparency, Responsibility, User-Focus, Sustainability, Technology, Lin et al. 2020) should be irrefutable assets for all national data infrastructures. A key focus must be on creating a digital data ecosystem that enables trustworthy, semantically interoperable data exchange while ensuring that data providers retain full, transparent, and traceable control over their use (e.g., as demonstrated by Pettenpohl et al. 2022).

To involve different stakeholders it is important to take into account different data cultures, which encompass various social, technical, and cultural characteristics, values, and practices that shape or determine how data are produced, generated, acquired, maintained, used, curated, stored, shared, and reused by individuals, organizations, governments, and societies (Oliver et al. 2023). Oliver and colleagues (2023) furthermore conclude that it is essential to recognize that diverse data cultures can coexist and compete across multiple levels and are inherently dynamic and normative in nature and that stakeholder engagement must create a trusting environment to accompany this process. Indigenous knowledge systems related to biodiversity must be recognized, respected, and preserved in ways that align with the values, protocols, and self-determination of the communities that hold them, even we are aware, that benefit returns are still unsolved issues (e.g., Silva-Morales and Navarrete-Díaz 2025). Here, project funding and good communication can encourage innovation and progress in the development of benefit returns, tools, and technologies, but ultimately, users need reliable infrastructures. Otherwise, stakeholders will stick with their own state-of-the-art and easier-to-control systems if community solutions appear unstable or compromised.

## Stakeholders’ technical demands

The benefits of building national biodiversity data infrastructures lie in the cross-domain knowledge sharing and community building as well as in collaborative creation of shared expertise (bioinformaticians, computer scientists, taxonomists, ecologists, education and training, and others), shared technology (e.g., tools and services), shared data, and shared data products (e.g., through GBIF hosted portals, https://www.gbif.org/hosted-portals). Under the umbrella of “together we can achieve more,” data mobilization, harmonization and tools, services, and workflow development are challenges that unite seemingly unrelated projects and actors across very different typologies. On the tool and service provider side, this requires skilled staff with computer literacy, standardization and documentation skills, and the ability to oversee the maintenance and upkeep of data management systems in relation to all aspects of the data lifecycle. Perhaps even more importantly, it requires understanding of the needs of the various stakeholders and how this translates into infrastructure and services. On the one hand, stakeholders in respective biodiversity data infrastructures provide this technical service; on the other hand, this can be promoted through stakeholder involvement, self-organization, and knowledge transfer. Dedicated and active experts must ensure that biodiversity data infrastructures, based on state-of-the-art and routine IT technology, are constantly updated, adapted, and expanded to meet the diverse and evolving needs of various users (Waterhouse et al. 2024). Requirements vary depending on the user and their computer literacy and technical skills, ranging, for example, from free and user-friendly software for data transfer, management, and analysis, to socially influenced requirements and incentives (e.g., visualizations) and scientific or conservation-motivated needs (e.g., tools for correct species identifications, e.g., Birnholtz and Bietz 2003, Griffiths 2009, Faniel and Zimmermann 2011). The adaptability is essential for effective biodiversity data management and necessitates experts or collaborations who are not only skilled with the newest hardware and software but also have a common vision, in-depth knowledge of evolving standards, robust data exchange systems, and the rapidly evolving world around technology (e.g., drones, AI, edge computing). National biodiversity data infrastructures are especially suited to provide shared resources, sometimes named as commons. Treloar and Woodford (2025) emphasized on the importance of emerging commons, which also need to be coordinated and linked even beyond national infrastructures, like the European Open Science Cloud (EOSC: https://eosc.eu/eosc-about/), the Australian Research Data Commons (ARDC: https://ardc.edu.au/), the International Virtual Observatory Alliance (IVOA: www.ivoa.net/), or the African Open Science Platform (AOSP: https://aospea.org/). Ongoing support, maintenance, and updates are crucial for the continuous data mobilization, long-term sustainability, and accessibility of national as well as international invaluable resources. A helpdesk serving as a central point of contact for queries would provide essential support and add significant value to connect the various groups and needs to the infrastructure community. Close collaboration and continuous feedback with users are imperative to ensure that the tools and services developed meet user needs. From users’ perspectives, biodiversity data infrastructures should provide services and workflows that meet the relevant requirements, as well as services that would be inefficient or unfeasible for individual partner institutions to provide and can only be realized through joint operation. Often, this requires the dedicated efforts of knowledgeable professionals who possess a deep understanding of their data, the databases they work with, and the respective communities they serve, yet also understand what it means to build “products” (i.e., the product mindset). Involving experts is essential, but their availability is often limited as there are only few highly qualified and motivated experts for these tasks. Given the wealth of data available, data integration, visualization, statistical analysis, or other analyses tools can be extremely useful for large-scale biodiversity monitoring and research (Turner et al. 2022). They may also serve to facilitate the qualitative assessment of data from different sources to determine biodiversity status and change and to detect sampling error and bias in observational datasets (Isaac et al. 2020, Turner et al. 2022). The implementation of services and tools, for example, data access and mobilization, and provision of software tools (e.g., Ampliseq-pipeline: Straub et al. 2020; ENA Data Submission Toolbox: Roncoroni et al. 2021; VAT-tool: Authmann et al. 2015, Beilschmidt et al. 2023, and others) can lead to high data infrastructure usage by previously unanticipated communities, for example, in the field of water analysis (Schürz et al. 2023). SBDI, for example, had to scale up resources when re-annotating and re-indexing large amounts of data. Furthermore, there is a great need for central cloud services with the capability to flexibly allocate and scale computing resources, such as for the recognition of duplicates, versioning, synonymy servers, synchronization of tools and services, data transformations with semantic tools, the use of temporary object storage, or the operation of own applications in the cloud.

National biodiversity data infrastructures vary in their development stages, with differences in the availability of key infrastructure components such as cloud storage. Infrastructures differ significantly in their ability to provide networking capabilities, including ontologies and taxonomies. Integrating diverse data sources, such as expert knowledge, laboratory data, and bioinformatics outputs, is a complex challenge. Institutional IT policies and formal regulations can also impose restrictions on tool use, such as software and services from large IT companies, which may conflict with national data protection regulations. For example, actors within individual organizations, such as public authorities, may not have the freedom to choose which software and services to use, particularly in relation to services of large IT companies. Furthermore, using free and open software without long-term support or a large user community is often deemed unacceptable by stakeholders.

## Stakeholder integration process

National biodiversity data infrastructures need to determine whether and how to implement a process of stakeholder integration. Stakeholder involvement in biodiversity data infrastructures can range from a set number of infrastructure providers determining which tools and services are made available (e.g., ARISE), to a flexible network system where stakeholders are involved not only in the submission and use of data, but also in the provision and development of tools and services (e.g., the Living Atlases community based on ALA open source software). Stakeholder integration can occur at various levels, ranging from simple registration for an online portal, to formalizing the process through actions like signing a data use and service agreement, an MoU, or becoming a partner or Principal Investigator (PI). The involvement of stakeholders can be predefined and fixed or an ongoing process. However, it should be clearly agreed that all parties recognize the provision and use of data, software and hardware, education, and training as a valuable and useful contribution. Where possible, collaboration should be based on principles, such as FAIR (Wilkinson et al. 2016), CARE (Carroll et al. 2020), and Transparency, Responsibility, User-Focus, Sustainability, Technology (TRUST, Lin et al. 2020). FAIR and CARE especially recognize aspects of preservation, whereas TRUST, in particular, emphasizes sustainability (Treloar and Woodford 2025). Where possible, collaboration should incorporate FAIR data tools and services (Lannom et al. 2020) and adhere to international standards (e.g., ABCD, Darwin Core, EML, and MIxS for biodiversity studies), as well as established practices and procedures.

**Figure 2:**
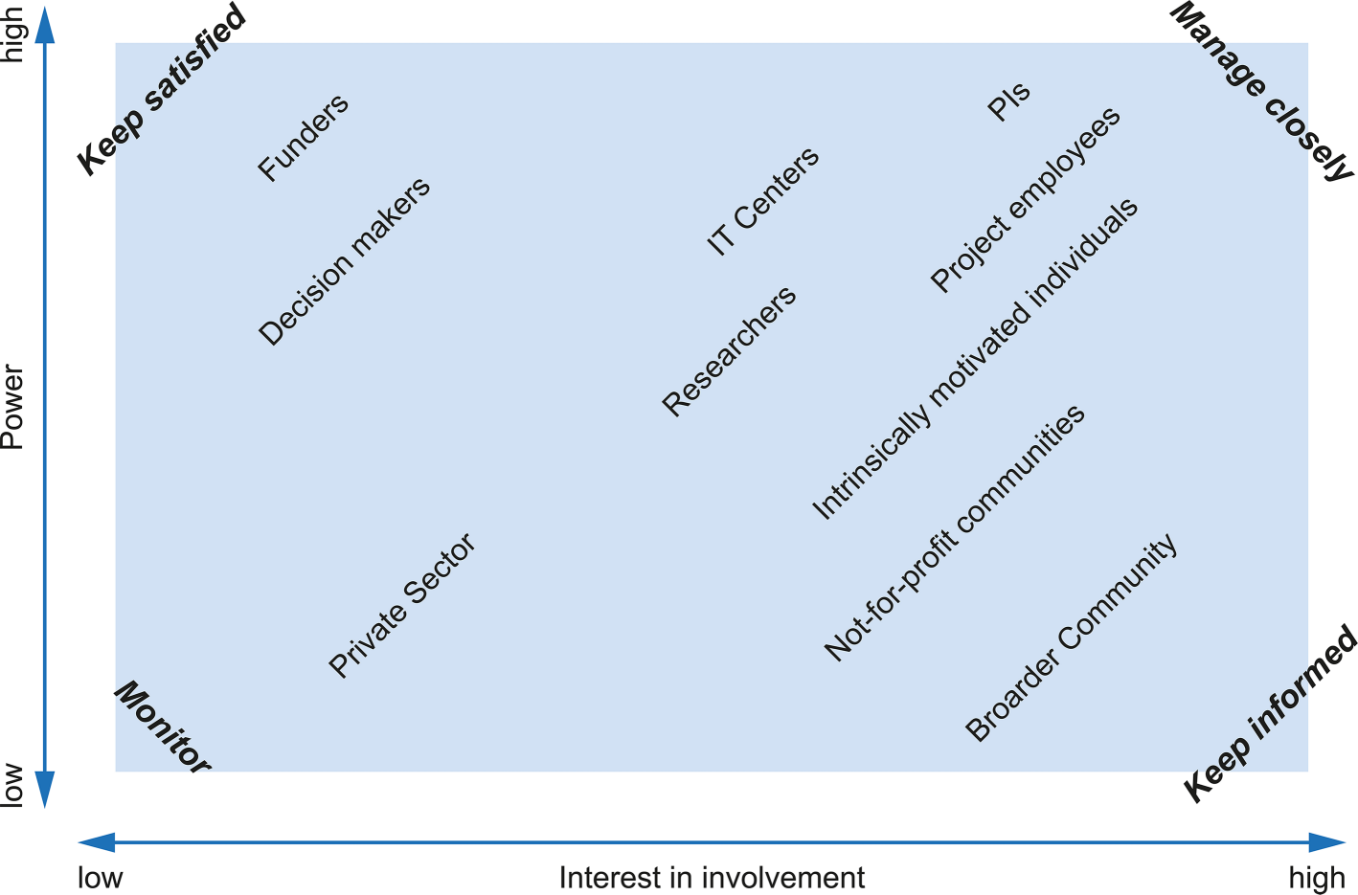
A power–interest grid for stakeholder management in national biodiversity data infrastructures.

In some cases, financial and personnel support can be beneficial both for individuals and for the collaborative process. In NFDI4Biodiversity, based on a flexible network system open for expansion on the data, tools, and services provisioning side, the strategy for stakeholder identification, integration, and engagement was to (1) engage a significant number of willing partners; (2) support them by identifying their needs; (3) assist them in addressing their challenges; and (4) make their data, tools, and services available (figure 2). The first contact points of national biodiversity data infrastructures are helpdesks or personal contacts. The NFDI4Biodiversity helpdesk with 1111 requests in 2024 was particularly important in many cases for standardizable data processing procedures that enable FAIR data publication in corresponding repositories. In the case of more complex requests, a discussion among the involved experts is needed to assess the anticipated added value for the respective stakeholders, other partners, and the infrastructure. The more the solution is positioned as exemplary, such as a blueprint for workflows in other organizations, the greater the likelihood that future requests will benefit from these developments. For that, selection criteria addressed the representability for (1) a certain taxonomic group, (2) a biodiversity data tool or technology, (3) a biodiversity database and analysis solution, and (4) the potential for transfer or general applicability to national biodiversity data activities or requests from service providers, data providers, or data centers . Other criteria related to staffing include the availability of qualified, motivated, and responsive contact persons with dedicated time allocation, who are willing and capable of taking on specific tasks. In many cases, small financial contributions, such as funding a few months of IT support or providing personnel support from experts for a short, predefined period, have resolved long-standing stakeholder issues. However, given the actual time and effort required for pilot projects, careful expectation management is essential for both simple and complex developments to avoid disappointments and unrealistic expectations.

Efforts to establish relationships between stakeholders help to group them, identify common activities and needs, categorize their participation levels, and assess the importance of each stakeholder. It is important to determine whether relationships are congruent or conflicting. Congruent relationships allow common interests to be realized more effectively, whereas conflicting relationships can jeopardize entire project goals. For national biodiversity data infrastructures, there should be more goal complementarities than conflicts to ensure that stakeholders remain invested in the infrastructures’ survival and further development. To this end, it is important to identify, integrate, and manage the needs of those willing to be involved.

## Stakeholder management

Stakeholder management, formalized by Freeman (1984), is embedded in corporate activities and can significantly influence strategies, processes, and performances concerning decision-making, relationship management, communication, innovation, and risk management (Pedrini and Ferri 2010). At the heart of good governance is genuine partner engagement, mutual trust, and satisfaction to ensure the network is fit for purpose and on target.

Many project management strategies emphasize the importance of prioritizing stakeholders using power–interest grids, which, however, are not static but engagement and priorities may change through time (figure 2). Nevertheless, these grids help visualize which stakeholders have both high interest and expectations, as well as the greatest impact on a project’s success. In the context of national biodiversity data infrastructures, PIs, their teams, and intrinsic motivations can lead individuals to become key stakeholders. These stakeholders should be satisfied with the ongoing work and results, and this should be continuously monitored as development progresses. Some powerful stakeholders may have limited interest or opportunity to participate actively in day-to-day operations. These stakeholders, such as funders or decision-makers, should be regularly updated and effectively managed to support the project’s goals. Data providers are immensely critical to the project’s success, contributing significantly to the availability and use of research data, tools, and services. They need to be informed and updated continuously, providing the appropriate level of detail to ensure engagement. Finally, low-interest, low-power stakeholders may find excessive communication unnecessary. For example, although the private sector is not yet eager to engage with national biodiversity data infrastructures, it should still be kept informed, as needed, to inform of the benefits of such engagement and potentially attract their involvement in the future.

Effective stakeholder management requires shared commitment and a culture of collaboration to create a comprehensive framework for capturing existing knowledge and identifying critical gaps (Özdemir et al. 2023). Stakeholders must demonstrate their commitment to compliance with governance structures, which must be consistently promoted and communicated. Clear governance structures, task distribution, knowledge sharing, prioritization, services, and bioinformatics development are key aspects of effective stakeholder management, ensuring the network remains fit for purpose and on target. However, continuous adaptation to changes in stakeholders’ roles and financial or personnel commitments may be required, which always demands a trade-off between forward-thinking commitments and administrative time to document changes. Feasibilities between tasks, financial, and human resources structured according to strategic importance, effort, staff capacity and timeframe, and attempts to strike a balance between different user groups are eminent tasks to ensure continuous stakeholder engagement. It may be advantageous to identify the core tasks and appoint a main contact person or a consultant for the respective tasks who manages the project control and, in some cases, the documentation requirements.

In NFDI4Biodiversity project, plans for different partner activities were developed that require additional funds or personnel support, based on the projects’ overall objectives and priorities. The project plans comprised predefined milestones, deliverables, responsibilities for people involved, and timelines to organize interdependent parts of the workflow. These project plans were updated as changes occurred (e.g., in human or financial resources or as the use cases were refocused, reviewed, and revised for various reasons). Clear but adaptive working plans are key to successful collaboration but have not always been possible. It is important to communicate that goals, roles, contacts, and ways of working with various stakeholders sometimes change over time. Often, details and stumbling blocks are only recognized in the course of a work process. As a result, some flexibility in project planning and execution is required, and even established goals and timelines shift due to resource constraints. This underscores the importance of effective management and detailed documentation. Changes in goals cannot be avoided, and in this context, some processes are quite simple and straightforward and can be resolved more quickly than initially anticipated, whereas other processes are significantly more time consuming and may result in delays. Caution is advised, as data mobilization can be a never-ending process, but one that requires clear, timely goals and interim results in addition to continuous, sufficient, and reliable funding. Temporary funding commitments of varying amounts, especially in times of severe budget cuts, can hinder progress. However, clear objectives and prioritization can alleviate this problem to some extent, as SBDI has demonstrated following a severe budget cut.

## Stakeholder communication

Stakeholder communication is important throughout all project phases (Canfield et al. 2024). It requires establishing and maintaining the network, not only through ongoing personal outreach but also when working in transdisciplinary teams with colleagues from various backgrounds, used to different terminologies, time availability, and constraints. Effective communication must ensure genuine partner engagement, mutual trust, and satisfaction among all. A common understanding of objectives, roles, and working methods must not only be developed and communicated, but also continuously revised, updated, and conveyed. Stakeholder engagement and integration can be achieved through activities open to the entire biodiversity community (e.g., newsletters, workshops, summer schools, online training materials, conference booths, helpdesks, and other outreach efforts) without requiring specialized commitments. For trust building and integrative cocreation, in-person meetings and regular participation in community-specific conferences, meetings, and workshops are crucial. Online meetings are great for factual exchanges and completing agreed-upon work. Stakeholder-specific conferences as well as various smaller project meetings have worked wonders in building community spirit among involved stakeholders. It is beneficial to designate a primary contact for respective tasks helping to navigate the project´s governance and documentation requirements. To optimize communication between the main active partners, a collaborative wiki platform and a ticket system set up by the central project coordination for project management and coordination are helpful to monitor and consolidate the different activities in the consortium and to develop project results, such as documentation and specifications, as a team. This provides information on the status, requirements, schedule, and resources required for each use case. For more distant partners, a website and regular newsletters, annual meetings, and face-to-face interactions are more useful, as the collaborative platform may prove too large and cumbersome for some of the stakeholders.

## Training, education, and legal advice

Data sharing is not necessarily a given in the field of ecology and biodiversity, as both research areas are traditionally known for their independent nature. Only recently has the greater potential of collaboration for common goals been increasingly emphasized, in contrast to within the research area of physics, for example, where data sharing and wide collaboration has long been the norm. As a result, the benefits of effective data management have not yet been fully incorporated into many research groups and undergraduate and graduate curricula. Biodiversity stakeholders exhibit varying levels of maturity in data management, so there is an urgent need to promote cultural change. This requires education, training, incentives, and streamlined data management processes so that a standard for FAIR data provision becomes commonplace. Broad-based collaboration, effective communication, the development of training materials, the provision of benefits, top-down as well as bottom-up incentives and motivation are crucial in this context. This may be beyond the scope of a national biodiversity data infrastructure alone, but requires collaboration at many different levels, or information and knowledge sharing when large consortia collaborate in an exemplary way. For example, discipline-specific consortia within Biodiversity Genomics Europe have formed to create the iBol Europe community, which provides best practices, training, workshops, and communication for the development of molecular-based European reference databases for species and research through a collegial knowledge platform (https://iboleurope.org/). GBIFs’ regional helpdesks and support offices as well as globally evolving data competence centers showcase how beneficial training and education can be achieved. On a different scale and in direct contact with the grassroots are the university IT support centers and libraries. They offer training on generic topics, and collaborate with regional, national, and international initiatives to make discipline-specific tools and training available to their students and researchers. The university flagship HeFDI (Hessian Research Data Infrastructures), for example, is offering generic RDM solutions and training, linked to the national biodiversity data infrastructure (e.g., see www.uni-marburg.de/en/hefdi/data-information-consulting/info-materials-and-publications; https://zenodo.org/communities/hefdi/records?q=&l=list&p=1&s=10&sort=newest).

YouTube/Vimeo videos, other social media content, and publications can serve as valuable sources of information (see, e.g., Röder et al. 2025). These self-paced digital training materials can complement digital, hybrid, and face-to-face workshops (e.g., Research Data Management for Biologists: https://github.com/NFDI4Biodiversity/nfdi4biodiversity-sle). Summer and winter schools may provide more valuable, personalized learning experiences, promote better networking among participants, and lead to a better understanding of data quality and its discrepancies or biases. A thorough understanding of the provenance of data used for scientific purposes in the field of biodiversity is essential for the effective integration of data from different sources, including legal considerations for handling highly proprietary or sensitive information. In particular, training in the use of cloud services and powerful data analysis tools for the biodiversity community is becoming increasingly important.

In the biodiversity sector, there is a significant need for legal knowledge about licensing, data ownership and data sharing, especially among participatory experts, scientists, taxonomists, and government agencies and authorities when data are generated in a proprietary way. The transfer of data from external sources into databases poses challenges for many downstream users, especially in terms of data protection. The legally correct handling of data remains an under-researched and unresolved issue in the context of biodiversity. Even when national biodiversity data infrastructures provide legal training, this does not necessarily reach grassroots data collectors, especially those involved in government monitoring programs who may be skeptical about data sharing. To overcome this challenge, easily accessible legal and data licensing advice for stakeholders is essential.

## Outlook

The term biodiversity dates to the mid-1980s (Tangley 1985, Sarkar 2021) and is commonly used today. Biodiversity combines previously very isolated research disciplines such as systematics, taxonomy, nature conservation, organismic ecology, and remote sensing as well as genetic research. Since then, the range of actors generating and using biodiversity data has expanded rapidly. New data formats have emerged, enabling new technologies such as remote sensing, eDNA, and AI-modelled information to answer entirely new research questions. At the same time, the rapid global loss of biodiversity urgently centers the need for open and accessible data, enabling this biodiversity data to be analyzed in different effective contexts. There is a high demand for monitoring and research in the field of biodiversity (e.g., to better understand organismal interactions between species, identify key species in ecosystems, and understand their roles in the context of ecosystem functions and host–vector analyses on different spatial and temporal scales). This has led to stakeholders feeling responsible not only for providing data, but also for standardization, scaling, and additional functionality to support biodiversity data for integrated analyses. Stakeholders develop new tools and services that support the mobilization of newly generated biodiversity data on mobile devices or automated sensor stations while continuing to record biodiversity using traditional methods. Thanks to pioneers in the field of biodiversity data networking, we can now build on tested, proven, robust concepts, tools, and experiences. The important challenge now is to build on this foundation and expand it with national baseline data, which is often lacking or only sporadically available for most organism groups in most countries. Furthermore, this needs to be linked to provide “long-tail” data and benefit from cooperation (Stahlman and Kouper 2024). Without this data, conservation efforts and political decisions, though well-intentioned, may be ineffective, jeopardizing the future protection of species. However, actions based on this information may help to minimize the impact of species loss, despite climate change, anthropogenic impacts on nature, and other threats.

## Conclusions

As outlined in Güntsch et al. (2024), stakeholders have increasingly established national biodiversity data infrastructures to act as hubs for biodiversity knowledge. These infrastructures provide tools and services for national stakeholder communities while also connecting to international biodiversity data networks in the sense of global open research commons (Treloar and Woodford 2025), for example, GBIF (e.g., Steinke et al. 2025), and OBIS. This supports evidence building by “long-tail” data (Stahlman and Kouper 2024) in a FAIR way. The stakeholders interested in these national biodiversity data infrastructures differ in terms of data, services, tools, and support offered. In all cases, however, a key commonality is that they add value through their role in data provision, networking, analysis, and archiving, and strengthen national data management activities related to biodiversity data as a whole. The result is support for activities at all scales, sectors, and levels of biodiversity expertise. The key considerations for effectively integrating stakeholders into national biodiversity data infrastructures include defining the stakeholder community and their benefits, grouping them appropriately, understanding their concerns and motivations, addressing their technical demands, establishing integration, management and communication strategies for their support, and identifying needs for education, training, and legal advice. These collaborative efforts enhance data mobilization and create value through automation, streamlined curation, archiving, interoperability, and advanced analytics for both basic and applied science, as well as biodiversity policy and governance. As mentioned in the report on the data revolution (IEAG 2014), we need cooperation and coordination to make the Sustainable Development Goals effective. This requires good cooperation between old and new data producers, the involvement of data users, and the development of ethical, legal, and statistical standards to improve data quality and protect against data misuse in a rapidly changing data ecosystem (IEAG 2014). Last but not least, this also requires sustainability not only in infrastructure maintenance but also in engagement, governance, and participation (e.g., see Treloar and Woodford 2025). With these recommendations, we aim to facilitate the integration of stakeholders in the development of national biodiversity data infrastructures, ensuring that the valuable data, tools, and services these stakeholders produce inform research and promote efficient implementation and management to achieve the Global Biodiversity Framework targets (GBF) and Sustainable Development Goals.
